# Commentary: Epileptic seizure clustering and accumulation at transition from activity to rest in GAERS rats

**DOI:** 10.3389/fneur.2024.1394248

**Published:** 2024-05-09

**Authors:** Péter Halász, Anna Szücs

**Affiliations:** ^1^Szentágothai Doctoral School, Semmelweis University, Budapest, Hungary; ^2^Institute of Behavioral Sciences, Semmelweis University, Budapest, Hungary

**Keywords:** absence epilepsy, burst-firing working mode of the thalamus, GAERS rats, behavioral state, NREM sleep

We have read with interest the paper of Tran et al. entitled “*Epileptic seizure clustering and accumulation at transition from activity to rest in GAERS rats*” published on 24th January 2024 in Frontiers in Neurology.

The authors describe the clustering and accumulation of seizures with spike-wave discharges in a time window of transition from activity to rest states in GAERS rats, a well-known model for absence epilepsy. They conclude that “these results point to mechanisms that control *behavioral states* as determining factors of seizure occurrence.”

While their findings on seizure clustering and predictors are interesting and warrant further analysis, we are surprised to note that for defining activity-rest states, they have not used the most obvious tool—electroencephalography. Hence, they may have missed the most important aspect of their finding in the generation of absence seizures. Specifically, the transition period from wakefulness to NREM sleep has been established as the “behavioral state” favoring the appearance of absences, as evidenced by previously cited works. The role of NREM sleep, the switch of the reticular nucleus of the thalamus to its burst-firing mode related to NREM, is known to be the “favorite” period of absences in humans. Thus, their question on “the behavioral state” that may promote absences has long been answered (1–3).

## Author contributions

PH: Writing – original draft, Writing – review & editing, Conceptualization. AS: Writing – original draft, Writing – review & editing.
